# Image Processing-Based Detection of Pipe Corrosion Using Texture Analysis and Metaheuristic-Optimized Machine Learning Approach

**DOI:** 10.1155/2019/8097213

**Published:** 2019-07-11

**Authors:** Nhat-Duc Hoang, Van-Duc Tran

**Affiliations:** ^1^Lecturer, Faculty of Civil Engineering, Institute of Research and Development, Duy Tan University, R.809–No.03 Quang Trung, Da Nang 550000, Vietnam; ^2^Lecturer, International School, Duy Tan University, 254 Nguyen Van Linh, Danang 550000, Vietnam

## Abstract

To maintain the serviceability of buildings, the owners need to be informed about the current condition of the water supply and waste disposal systems. Therefore, timely and accurate detection of corrosion on pipe surface is a crucial task. The conventional manual surveying process performed by human inspectors is notoriously time consuming and labor intensive. Hence, this study proposes an image processing-based method for automating the task of pipe corrosion detection. Image texture including statistical measurement of image colors, gray-level co-occurrence matrix, and gray-level run length is employed to extract features of pipe surface. Support vector machine optimized by differential flower pollination is then used to construct a decision boundary that can recognize corroded and intact pipe surfaces. A dataset consisting of 2000 image samples has been collected and utilized to train and test the proposed hybrid model. Experimental results supported by the Wilcoxon signed-rank test confirm that the proposed method is highly suitable for the task of interest with an accuracy rate of 92.81%. Thus, the model proposed in this study can be a promising tool to assist building maintenance agents during the phase of pipe system survey.

## 1. Introduction

In high-rise building maintenance, an important objective is concerned with the integrity of the water supply system and prevention of water contamination. Cast iron is widely used in water supply and waste disposal systems due to the advantage of high strength. Since stainless steel pipes often fall out of favor in domestic pipework because of their high expenses [1], corrosion is a widely observed type of structural damage.

**Figure 1:**
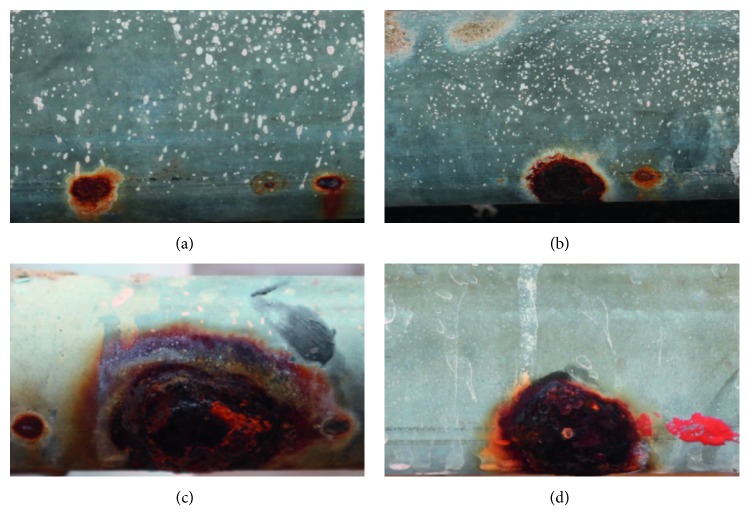
Corroded areas on pipe surface.

Corrosion (see Figure 1) can be defined as a chemical process caused by chemical and electrochemical reactions. This phenomenon is typically observed in environmental conditions featuring a high level of moisture. There are different kinds of corrosion such as general corrosion which occurs as uniformly distributed nonprotective flakes of rust and pitting which is a localized point of corrosive attack [2]. Corrosion brings about the destruction of metal pipework surface and consequently leads to reduction in pipe service life and increase in building maintenance cost [3]. In certain case, this defect may strongly affect the health of building occupants due to deterioration of water quality. Thus, corrosion should be identified timely by means of periodic surveys to ensure the integrity of pipe systems and establish cost-effective maintenance strategies.

In Vietnam as well as in many other countries, manual methods performed by human inspectors are commonly employed for condition assessment of water supply/waste disposal systems. As clearly pointed out by Liu et al. [4] and Atha and Jahanshahi [5], these manual approaches are labor intensive and time consuming. Corroded regions can be neglected in positions of pipe system that are difficult to reach and observe visually. Moreover, the processes of data processing and reporting are also very tedious for human technicians. Therefore, there is a practical need to come up with a more productive and accurate method of pipe condition survey.

Although there is a wide range of existing pipe inspection approaches (such as magnetic flux leakage, ultrasonic testing, and external corrosion direct assessment), all of these methods have limitations including high equipment cost, restricted range of inspection, and incapability of detecting small pitting regions [3]. Considering the large amount of pipe systems needed to be surveyed and the limited access to sophisticated equipment in developing countries, there is an urgent need for a productive and low-cost solution for periodic surveys of pipe system condition. Recently, digital image processing has gained a great attention within the field of structural heath monitoring [6, 7].

Particularly, image processing techniques can be effectively employed to investigate the outer surface for detecting defects on pipes or other metal structures including corrosion and cracks [8]. Itzhak et al. [9] relied on statistic measurement of image pixels to quantify pitting corrosion. Choi and Kim [10] identified corrosion based on the morphology of the corroded surface; features of image color, texture, and shape are employed for corrosion recognition. A model for classifying corroded and noncorroded surfaces using texture descriptors obtained from gray level co-occurrence matrix and image color has been proposed in Medeiros et al. [11].

A method based on watershed segmentation has been employed in [12] for rating of corrosion defects; the percentage area of corroded region was used for determining the grade of defects. Idris and Jafar [13] used image filter-based image enhancement and neural network for corrosion inspection. Son et al. [14] proposed a model based on decision tree algorithm for identifying rusted surface area of steel bridge. A model based on image color analysis and K-means clustering for bridge rust identification has been constructed and verified by Liao and Lee [15].

Petricca et al. [16] compared standard computer vision techniques and deep neural network for rust and nonrust detection. Deep neural networks have also been employed for corrosion detection by Liu et al. [4] and Atha and Jahanshahi [5]. Safari and Shoorehdeli [17] applied artificial neural network, Gabor filter, and entropy filter for pipe defect detection. Cheriet et al. [18] incorporated expert knowledge and field data to construct a knowledge-based system for assessing corrosive damage on metallic pipe conduits. Gibbons et al. [19] relied on a Gaussian mixture model for probabilistic classification of corroded and noncorroded areas. Bondada et al. [3] detected and quantitatively assessed corrosion damages on pipelines by computing the mean of saturation value of image pixels; by image analysis, the corroded areas on pipelines can be segmented.

From the above literature, it can be seen that image processing and machine learning have been a feasible alternative for replacing the tedious process of manual survey. Based on a recent review work of Ahuja and Shukla [20], there is an increasing trend of applying computer vision techniques for corrosion detection. Moreover, due to the importance of the research theme, exploring other image processing and machine learning methods used for pipe corrosion detection can be highly meaningful in both academic and practical aspects.

As reported in the literature, although image texture analysis has been applied, few previous studies have employed a combination of image texture descriptors for pipe corrosion recognition. Hence, this study is an attempt to fill this gap in the literature by proposing a method used for analyzing texture of water pipe surface that integrates statistical measurement of color channels, gray-level co-occurrence matrix, and gray-level run length matrix. Based on the features extracted by the above texture descriptors, the support vector machine (SVM) [21] is employed to categorize image samples into two classes: noncorrosion (negative) and corrosion (positive). SVM is utilized in this study due to the fact that it has been confirmed to be a robust tool for pattern classification in various studies [22–24]. In addition, to optimize the training process of SVM-based corrosion detection model, differential flower pollination (DFP) metaheuristic is employed. A dataset consisting of 2000 image samples has been collected to train and verify the proposed method.

The rest of the study is organized as follows.  Section 2 reviews the research material and methods used to construct the water pipe corrosion detection approach.  Section 3 reports experimental results and discussions.  Section 4 provides several concluding remarks of this study.

## 2. Material and Methods

### 2.1. Image Texture Analysis

Identifying corroded areas based on two-dimensional image samples is a challenging task due to the complex and deceptive features of pipe surfaces containing various irregular objects such as dirt and paints. Therefore, using information provided by one pixel is definitely not sufficient for corrosion detection. It is because a pixel having similar color values can belong to both categories of noncorrosion and corrosion. Hence, texture information extracted from a certain region of pipe surface can be used for recognizing the defect of interest. This section of the study describes the employed texture descriptors used for computing the features of water pipe surface.

#### 2.1.1. Statistical Properties of Color Channels

Herein, the statistical properties of three color channels (red, green, and blue) of an image sample can be employed to represent image texture. Thus, an image is described in a RGB color space. It is noted that besides RGB, there are other color spaces such as HSV which can also be useful in the task of corrosion detection. However, in this study, we rely on the original RGB color model obtained from the employed digital camera. Let *I* be a variable representing the color levels of an image sample. The first-order histogram *P*(*I*) is calculated as follows [25]:

(1) $$\begin{array}{c} P_{\text{c}}\left(I\right)=\frac{N_{I,\text{c}}}{W\times H}, \end{array}$$

where c denotes a color channel, *N*_*I*,c_ is the number of pixels with intensity value *I* of the channel c, and *H* and *W* represent the height and width of an image sample, respectively.

Thus, the mean (*μ*_c_), standard deviation (*σ*_c_), skewness (*δ*_c_), kurtosis (*η*_c_), entropy (*ρ*_c_), and range (Δ_c_) of color value are calculated as follows:

(2) $$\begin{array}{c} \mu_{\text{c}}=\overset{\text{NL}-1}{\underset{i=0}{\sum }}I_{i,\text{c}}\times P_{\text{c}}\left(I\right),\\ \sigma_{\text{c}}=\sqrt{\overset{\text{NL}-1}{\underset{i=0}{\sum }}{\left(I_{i,\text{c}}-\mu_{\text{c}}\right)}^{2}\times P_{\text{c}}\left(I\right),}\\ \delta_{\text{c}}=\frac{\sum_{i=0}^{\text{NL}-1}{\left(I_{i,\text{c}}-\mu_{\text{c}}\right)}^{3}\times P_{\text{c}}\left(I\right)}{\sigma_{\text{c}}^{3}},\\ \eta_{\text{c}}=\frac{\sum_{i=0}^{\text{NL}-1}{\left(I_{i,\text{c}}-\mu_{\text{c}}\right)}^{4}\times P_{\text{c}}\left(I\right)}{\sigma_{c}^{4}},\\ \rho_{\text{c}}=-\overset{\text{NL}-1}{\underset{i=0}{\sum }}P_{\text{c}}\left(I\right)\;\times \;\;\;\log_{2}\left(P_{\text{c}}\left(I\right)\right),\\ \Delta_{\text{c}}=\text{Max}\left(I_{\text{c}}\right)-\text{Min}\left(I_{\text{c}}\right), \end{array}$$

where NL = 256 denotes the number of discrete color values.

#### 2.1.2. Gray-Level Co-Occurrence Matrix (GLCM)

The GLCM [26] is also a commonly used texture descriptor. To employ this technique, a color image must first be converted to a gray scale one. The GLCM discriminates different image textures based on the repeated occurrence of some gray-level patterns existing in the texture [27]. Let *δ*=(*r*, *θ*) be a vector in the polar coordinates of an image sample. For each *δ*, the joint probability of the pairs of gray levels that occur at the two points separated by the relationship *δ* is computed [28]. This joint probability is compactly displayed in a GLCM *P*_*δ*_ within which *P*_*δ*_(*i*, *j*) represents the probability of the two gray levels of *i* and *j* occurring according to *δ*. The original *P*_*δ*_(*i*, *j*) is often normalized via the following equation:

(3) $$\begin{array}{c} P_{\delta }^{N}\left(i,j\right)=\frac{P_{\delta }\left(i,j\right)}{S_{\text{P}}}, \end{array}$$

where *P*_*δ*_^*N*^(*i*, *j*) denotes the normalized GLCM and *S*_P_ is the number of pixels.

Based on the suggestion of Haralick et al. [29], four GLCMs with *r* = 1 and *θ* = 0°, 45°, 90°, and 135° can be established. Accordingly, angular second moment (AM), contrast (CO), correlation (CR), and entropy (ET) for each matrix can be computed to serve as texture descriptors as follows [28, 29]:

(4) $$\begin{array}{c} \text{AM}=\overset{N_{\text{g}}}{\underset{i=1}{\sum }}\overset{N_{\text{g}}}{\underset{j=1}{\sum }}P_{\delta }^{N}{\left(i,j\right)}^{2},\\ \text{CO}=\overset{N_{\text{g}}-1}{\underset{k=0}{\sum }}k^{2}\underset{\left|i-j\right|=k}{\overset{N_{\text{g}}}{\underset{i=1}{\sum }}}\overset{N_{\text{g}}}{\underset{j=1}{\sum }}P_{\delta }^{N}\left(i,j\right),\\ \text{CR}=\frac{\sum_{i=1}^{N_{\text{g}}}\sum_{j=1}^{N_{\text{g}}}i\times j\times P_{\delta }^{N}\left(i,j\right)-\mu_{X}\mu_{Y}}{\sigma_{X}\sigma_{Y}},\\ \text{ET}=-\overset{N_{\text{g}}}{\underset{i=1}{\sum }}\overset{N_{\text{g}}}{\underset{j=1}{\sum }}P_{\delta }^{N}\left(i,j\right)\log\left(P_{\delta }^{N}\left(i,j\right)\right), \end{array}$$

where *N*_g_ is the number of gray-level values and *μ*_*X*_, *μ*_*Y*_, *σ*_*X*_, and *σ*_*Y*_ are the means and standard deviations of the marginal distribution associated with a normalized GLCM [29].

#### 2.1.3. Gray-Level Run Lengths (GLRL)

GLRL is a texture description method proposed by Galloway [30]. This method is highly effective in discriminating textures featuring different fineness and has been successfully applied in various fields of study [31, 32]. It is because GLRL is constructed based on the fact that relatively long gray-level runs are observed more frequently in a coarse texture and a fine texture typically has more short runs [33]. A run-length matrix *p*( *i · j* ) in a certain direction is defined as the number of times that a run length *j* of gray level *i* is observed [30].

Using this matrix, the short run emphasis (SRE), long run emphasis (LRE), gray-level nonuniformity (GLN), run length nonuniformity (RLN), and run percentage (RP) [30, 33] can be computed. Additionally, Chu et al. [34] put forward the indices of low gray-level run emphasis (LGRE) and high gray-level run emphasis (HGRE). Dasarathy and Holder [35] proposed to compute the short run low gray-level emphasis (SRLGE), short run high gray-level emphasis (SRHGE), long run low gray-level emphasis (LRLGE), and long run high gray-level emphasis (LRHGE). The above indices are summarized in Table 1. It is noted that one run length matrix is computed for each of direction in the set of {0°, 45°, 90°, 135°} and each matrix results in 11 GLRL-based features. Therefore, the total number of features obtained from GLRL matrices is 11 × 4 = 44.

### 2.2. Computational Intelligence Methods

#### 2.2.1. Support Vector Machine (SVM)

**Figure 2:**
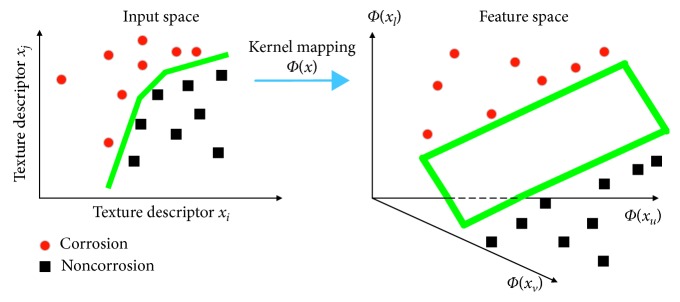
The data classification process of a SVM model.

SVM, described in [21], is a robust pattern recognition method established on the theory of statistical learning. Given the task at hand is to classify a set of input feature *x*_*k*_ into two categories of *y*_*k*_ = −1 (noncorrosion) and *y*_*k*_ = +1 (corrosion), a SVM model constructs a decision surface that separates the input space into two distinctive regions characterizing the two different two categories. The SVM algorithm aims at identifying a decision boundary so that the gap between classes is as large as possible [36]. In addition, SVM employs the kernel trick to convert a nonlinear classification task into a linear one. A SVM model first maps the input data from the original space to a high-dimensional feature space within which the data can be separated by a hyperplane (see Figure 2).

The SVM training process can be formulated as the following constrained optimization problem [36]:

(5) $$\begin{array}{c} \begin{array}{cc} \text{minimize} & J_{\text{p}}\left(w,e\right)=\frac{1}{2}w^{T}w+c\frac{1}{2}\overset{N}{\underset{k=1}{\sum }}e_{k}^{2}\\ \text{subjected to} & y_{k}\left(w^{T}\varphi \left(x_{k}\right)+b\right)\geq 1-e_{k},\;k=1,...,N,\;\;e_{k}\geq 0, \end{array} \end{array}$$

where *w* ∈ *R* ^*n*^ is a normal vector to the classification hyperplane and *b* ∈ *R* is the model bias; *e*_*k*_ ≥ 0 is called a slack variable; c denotes a penalty constant; and *φ*(*x*) is a nonlinear mapping from the input space to the high-dimensional feature space.

During the construction of a SVM model, it is not required to obtain the explicit form of *φ*(*x*). Instead of that, only the dot product of *φ*(*x*) in the input space is required and expressed via a kernel function shown as follows:

(6) $$\begin{array}{c} K\left(x_{k},x_{l}\right)=\varphi {\left(x_{k}\right)}^{T}\varphi \left(x_{l}\right). \end{array}$$

The radial basis function (RBF) kernel function [37] is often employed for data classification; its functional form is given below:

(7) $$\begin{array}{c} K\left(x_{k},x_{l}\right)=\exp\left(-\frac{{\left\|x_{k}-x_{l}\right\|}^{2}}{2\sigma^{2}}\right), \end{array}$$

where *σ* is a free parameter.

Accordingly, a SVM model used for data classification is given compactly as follows:

(8) $$\begin{array}{c} y\left(x_{l}\right)=\text{sign}\left(\overset{\text{SV}}{\underset{k=1}{\sum }}\alpha_{k}y_{k}K\left(x_{k},x_{l}\right)+b\right), \end{array}$$

where *α*_*k*_ denotes the solution of the dual form of the aforementioned constrained optimization. SV is the number of support vectors which is the number of *α*_*k*_ > 0.

#### 2.2.2. Differential Flower Pollination (DFP)

As shown in the previous section, the model training and prediction phases of a SVM model depend on a proper selection of its hyperparameters including the penalty coefficient (c) and the kernel function parameter (*σ*). The first hyperparameter affects the penalty imposed on data samples deviating from the established decision surface; the later hyperparameter specifies the smoothness of the decision surface. Since this problem of hyperparameter selection can be formulated as an optimization problem [38–40], this study employs the DFP metaheuristic to optimize the model training phase of SVM.

DFP, proposed in [41], is a population-based metaheuristic that combines the advantages of the standard algorithms of differential evolution (DE) [42] and flower pollination algorithm (FPA) [43]. The employed hybrid metaheuristic consists of three main steps: initialization of population members, alteration of member locations, and cost function evaluation. Each member of the DFP metaheuristic is presented as a numerical vector consisting of the two SVM hyperparameters. In the first step, all population members are randomly generated within the feasible domain. In the second step, the location of population members is altered by local and global search phases. In the next step, the cost function of each member is computed and a greedy selection operator is performed to update the location of the DFP's population.

The second step of the DFP includes the FPA-based global pollination operator and the DE-based local pollination operator. A switching probability *p*=0.8 is used to govern the frequencies of these two operators [43]. The FPA-based global pollination and the DE-based local pollination operators are presented as follows:

(i) The FPA-based global pollination:
  (9) $$\begin{array}{c} X_{i}^{\text{trial}}=X_{i}^{{\text{g}}_{\text{DFP}}}+L\cdot \left(X_{i}^{{\text{g}}_{\text{DFP}}}-X_{\text{best}}\right), \end{array}$$
  where g is the index of the current generation, *X*_*i*_^trial^ is a trial solution, *X*_*i*_^g_DFP_^ denotes a solution of the current population, *X*_best_ represents the best solution, and *L* denotes a random number generated from the Lévy distribution [43].
(ii) The DE-based local pollination modifies the current member by creating a mutated flower and a crossed flower according to the following equations:
  (a) Creating a mutated flower:
    (10) $$\begin{array}{c} X_{i,{\text{g}}_{\text{DFP}}}^{\text{mutated}}=x_{r1,{\text{g}}_{\text{DFP}}}+F\cdot \left(x_{r2,{\text{g}}_{\text{DFP}}}-x_{r3,{\text{g}}_{\text{DFP}}}\right), \end{array}$$
    where *r*1, *r*2, and *r*3 are three random integers and *F* denotes a mutation scale factor which is drawn from a Gaussian distribution with the mean = 0.5 and the standard deviation = 0.15 [41].
  (b) Creating a crossed flower:
    (11) $$\begin{array}{c} X_{j,i,{\text{g}}_{\text{DFP}}+1}^{\text{crossed}}=\left\{\begin{array}{cc} X_{j,i}^{\text{mutated}}, & \text{if}\;\;r\text{ and }j\leq \text{Cr or}\;\;j=\text{rnb}\left(i\right),\\ X_{j,i,{\text{g}}_{\text{DFP}}}, & \text{if}\;\;r\text{ and }j>\text{Cr and}\;\;j\neq \text{rnb}\left(i\right), \end{array}\right. \end{array}$$
    where Cr = 0.8 is the crossover probability [44].

### 2.3. Collected Image Samples

Because SVM is a supervised machine learning algorithm, a dataset consisting of 2000 image samples of pipe surface with the ground truth label has been collected to construct the SVM-based corrosion detection model. It is proper to note that the numbers of image samples in the two labels of noncorrosion (negative class) and corrosion (positive class) are both 1000. The digital image samples have been collected during surveys of several high-rise buildings in Danang city (Vietnam). The used digital camera is the 18-megapixel resolution Canon EOS M10, and the images were manually acquired by human inspectors.

**Figure 3:**
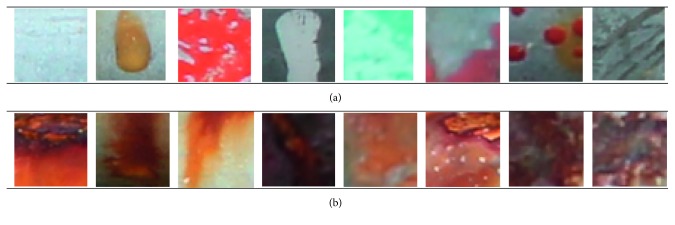
The collected image samples: (a) noncorrosion class and (b) corrosion class.

Accordingly, image samples of the two labels of noncorrosion (label = −1) and corrosion (label = +1) have been prepared for SVM-based classification process. In order to accelerate the texture computation process, the size of image samples has been set to be 50 × 50 pixels. Hence, image cropping operation is performed to generate the image samples used to train the SVM model. The collected image set is illustrated in Figure 3.

### 2.4. Proposed Hybridization of Image Processing and Metaheuristic-Optimized SVM for Pipe Corrosion Detection

**Figure 4:**
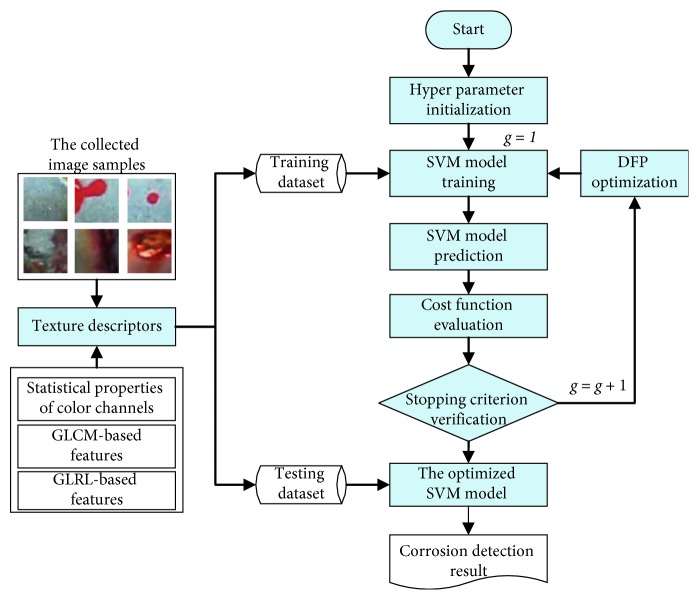
The proposed MO-SVM-PCD model used for pipe corrosion detection.

This section of the study describes the structure of the newly developed hybrid model of image processing and metaheuristic-optimized SVM for pipe corrosion detection. The proposed model, named as MO-SVM-PCD, is a combination of image texture analysis and a metaheuristic-optimized machine learning approach. As mentioned earlier, the statistical measurements of color channels, GLCM, and GLRL are used to extract texture-based features from image samples. The hybrid model relies on SVM to classify data samples into the categories of noncorrosion and corrosion. In addition, the DFP metaheuristic is employed to optimize the SVM-based training and prediction phases. The overall structure of the MO-SVM-PCD model is shown in Figure 4. The model structure can be divided into two separated modules: computation of image texture and data classification based on SVM. The first module is constructed in Visual C#.NET; the second module is developed in MATLAB.

Within the first module, the image texture descriptors based on statistical analysis of color channels, GLCM, and GLRL compute numerical features from image samples. For each of the three color channels (red, green, and blue), six statistical measurements of mean, standard deviation, skewness, kurtosis, entropy, and range are calculated. Hence, the total number of numerical features extracted from the aforementioned statistical indices of an image sample is 6 × 3 = 18. Subsequently, the group of features extracted from the four co-occurrence matrices corresponding to the directions of 0°, 45°, 90°, and 135° is computed. Because four indices of the angular second moment, contrast, correlation, entropy are acquired from one co-occurrence matrix, the total number of GLCM-based features is 4 × 4 = 16.

In addition, each GLRL matrix yields 11 properties of SRE, LRE, GLN, RLN, RP, LGRE, HGRE, SRLGE, SRHGE, LRLGE, and LRHGE. Thus, as stated earlier, the number of GLRL-based features is 4 × 11 = 44. Accordingly, each image sample is characterized by a feature vector having 18 + 16 + 44 = 78 elements. This module can compute texture of one image for illustration purpose and can extract features from a batch of image samples to construct the training and testing numerical datasets.

When the module of feature computation is accomplished, a dataset consisting of 2000 data samples and 78 input features is ready for further analysis. This dataset has two class outputs: −1 meaning noncorrosion (negative class) and +1 meaning corrosion (positive class). In addition, for standardizing the data ranges and enhancing the data modeling process, the numerical dataset is preprocessed by the Z-score data normalization [45]. The equation of the Z-score data normalization is given as follows:

(12) $$\begin{array}{c} X_{\text{ZN}}=\frac{X_{\text{o}}-m_{X}}{s_{X}}, \end{array}$$

where *X*_o_ and *X*_ZN_ represent an original and a normalized input variable, respectively, and *m*_*X*_ and *s*_*X*_ denote the mean and the standard deviation of the original input variable, respectively.

Subsequently, the normalized dataset is randomly divided into two subsets: a training set (70%) and a testing set (30%). The first data subset is employed for model training; the later data subset is reserved for model testing. The training dataset is employed by the SVM-based data classification module to generalize a corrosion recognition model. In addition, DFP is utilized to finetune the SVM model hyperparameters including the penalty coefficient and the RBF kernel parameter. It is worth mentioning that the SVM model operates via the help of the MATLAB's Statistics and Machine Learning Toolbox [46]; in addition, the DFP and the hybridization of DFP and SVM model have been constructed in MATLAB by the authors.

As shown in Figure 4, the two SVM hyperparameters are randomly initialized at the first generation (*g*=1). Using the local and global pollination operators, the DFP algorithm gradually guides the population of SVM hyperparameters to explore the search space and identify better solutions. Based on the guidance of parameter setting in previous studies [44, 47], the population size and the number of DFP searching generations are selected to be 12 and 100. The feasible domain of the SVM's penalty coefficient and kernel parameter is [1, 100] and [0.1, 100], respectively. In the phase of solution evaluation, the quality of each member in the population is appraised via the following cost function:

(13) $$\begin{array}{c} f_{\text{CF}}=\frac{\sum_{k=1}^{K}2/\left({\text{PPV}}_{k}+{\text{NPV}}_{k}\right)}{K}, \end{array}$$

where *K* = 5 denotes the number of data folds and PPV and NPV are the positive predictive value and the negative predictive value. PPV and NPV are employed to express the model performance associated with a set of SVM hyperparameters.

PPV and NPV are computed according to the following equations [48]:

(14) $$\begin{array}{c} \text{PPV}=\frac{\text{TP}}{\text{TP}+\text{FP}},\\ \text{NPV}=\frac{\text{TN}}{\text{TN}+\text{FN}}, \end{array}$$

where TP, TN, FP, and FN are the true positive, true negative, false positive, and false negative values, respectively.

It is noted that to compute the model's cost function, a *K*-fold cross validation process with *K* = 5 is employed. Using this cross fold validation, the original dataset is separated into 5 mutually exclusive subsets. Accordingly, the SVM model training and evaluation is repeated 5 times. In each time, 4 subsets are utilized for model training and one subset is used for model validation. The overall model performance is obtained via averaging predictive outcomes of the 5 data folds. This process has been proved to be a robust method for model hyperparameter selection [49]. Notably, in each generation, based on the computed cost function, the location of population members is updated and the stopping criterion is checked to verify whether the current generation number exceeds the allowable value. If the stopping criterion is met, the DFP-based optimization process terminates and the optimized SVM model is ready to predict corrosion status for novel image samples.

## 3. Experimental Results and Discussion

As stated earlier, the dataset featuring 2000 samples and 78 image texture variables has been separated into the training and testing subset. The training and testing subsets occupy 70% and 30% of the original dataset, respectively. The first subset is used for model training. The second subset is employed for testing the model predictive capability when it predicts corrosion status of novel image samples which has not been encountered in the training subset. Moreover, to reliably assess the model performance and to diminish the randomness caused by the data sampling process, this research work has conducted a random subsampling of the original dataset consisting of 20 runs. In each run, 30% of the data is randomly extracted to constitute the testing subset; the rest of the data is used for model training. Accordingly, the overall model performance is reliably evaluated by averaging prediction results obtained from the repeated data sampling.

In addition to the aforementioned PPV and NPV, the classification accuracy rate (CAR), recall, and F1 score are also used for expressing the model's predictive accuracy. These indices are computed as follows [50]:

(15) $$\begin{array}{c} \text{classification accuracy rate: CAR}=\frac{\text{TP}+\text{TN}}{\text{TP}+\text{TN}+\text{FP}+\text{FN}}\times 100\%,\\ \text{recall}=\frac{\text{TP}}{\text{TP}+\text{FN}},\\ \text{F}1\text{ score}=\frac{2\text{TP}}{2\text{TP}+\text{FP}+\text{FN}}, \end{array}$$

where TP, TN, FP, and FN are the true positive, true negative, false positive, and false negative values.

**Figure 5:**
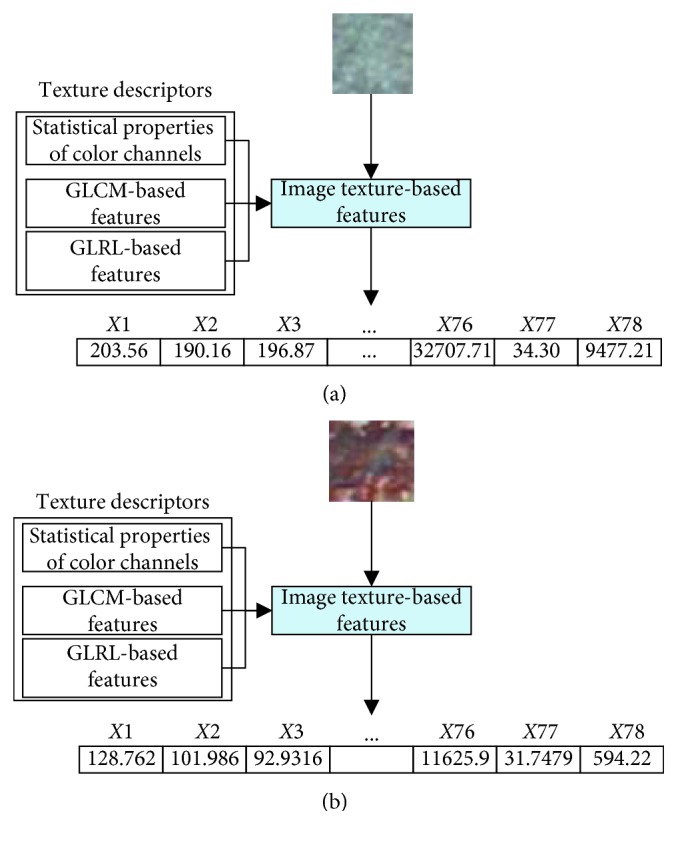
Illustration of the feature extraction process: (a) a noncorrosion case and (b) a corrosion case.

**Figure 6:**
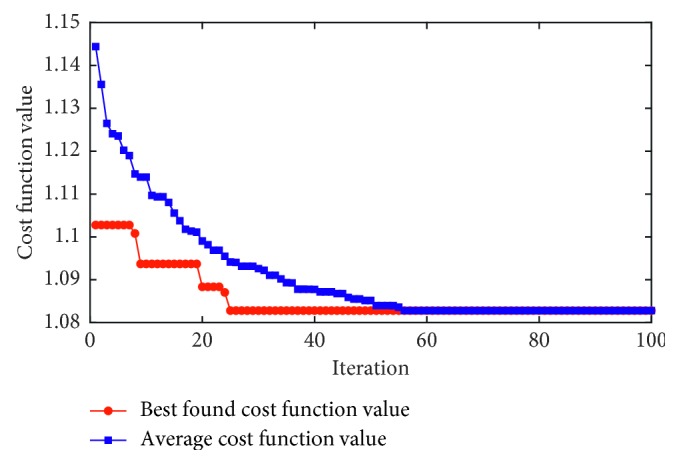
The DFP optimization process.

Demonstration of the feature extraction phase which computes image sample texture is provided in Figure 5. Herein, for each image sample, 78 features representing the statistical measurements of image colors, GLCM, and GLRL are attained and used for data classification purpose. In addition, the evolutionary process of the DFP metaheuristic-based SVM model optimization is illustrated in Figure 6 which shows the best and the average cost function values in each generation. The optimal values of the penalty coefficient and the RBF kernel function parameter are found to be 4.30 and 8.86 with the best cost function = 1.08.

The performance of the MO-SVM-PCD in the training and testing phases is reported in Table 2. As shown in this table, the proposed model has attained good predictive accuracy in both phases with CAR >90%. In detail, the MO-SVM-PCD has achieved CAR = 91.17%, PPV = 0.91, recall = 0.92, NPV = 0.92, and F1 score = 0.91 in the testing phase. There is a focus on the MO-SVM-PCD performance in the testing phase because this reflects the generalization capability of the model.

**Figure 7:**
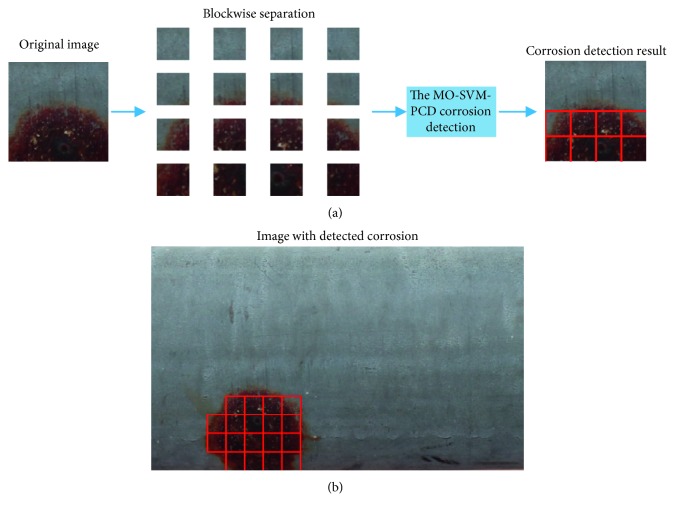
The MO-SVM-PCD-based corrosion detection result: (a) blockwise image separation process and (b) detection outcome with a large-sized image sample.

In addition, corrosion detection based on the MO-SVM-PCD for a large-sized image samples can be achieved via a blockwise image separation process. This image separation process is illustrated in Figure 7(a). In this figure, each block corresponds to a sample having the size of 50 × 50. The classification result for the entire image is carried out by combining the MO-SVM-PCD-based corrosion detection for each image block (see Figure 7(b)). The computational time required to classify one image block is about 4 seconds; therefore, the corrosion detection of the whole large-sized image (800 × 600 pixels) requires about 768 seconds. It is noted that the detected positive class (corrosion class) samples are highlighted by red squares. As can be seen from this figure, the proposed approach can achieved relatively good classification result. Nevertheless, several positive samples located in the boundary of the corroded area have not been identified correctly.

Furthermore, to better demonstrate the prediction capability of the newly constructed MO-SVM-PCD employed for detecting metal pipe corrosion, its performance has been compared to that of the least squares support vector machine (LSSVM) [51], classification tree (CTree) [52], backpropagation artificial neural network (BPANN) [53], and convolutional neural network (CNN) [54]. The reason for the selection of these benchmark models is that they have been confirmed to be capable methods for pattern classification by previous studies [5, 40, 55–57].

The LSSVM model is programmed in MATLAB by the authors; its tuning parameters including the regularization coefficient and kernel function parameter are also automatically identified by the DFP metaheuristic. The CTree is developed by the built-in functions provided in the MATLAB Statistics and Machine Learning Toolbox [46]. The BPANN model is programmed in MATLAB environment by the authors. Via experiment, the suitable parameter of minimum leaf size of the employed CTree model has been found to be 2. Based on the suggestions of Heaton [58] and several trial-and-error runs, the number of neuron in the hidden layer of the BPANN model is selected to be 2 × *N*_I_/3 + *N*_O_ = 54, where *N*_I_ = 78 is the number of input features and *N*_O_ = 2 is the number of class labels. Moreover, the learning rate and the number of training epochs of the neural network are set to be 0.1 and 1000, respectively.

In addition, the CNN model employed for corrosion detection is constructed by the MATLAB image processing toolbox [59]; the stochastic gradient descent with momentum (SGDM) and mini-batch mode are used in the model training phase. Via experimental runs, the appropriate configuration of the deep learning method is as follows: input image size is 50 × 50 pixels. The number of convolution layers is 4. The sizes of the filters are 20 × 20, 16 × 16, 8 × 8, and 4 × 4 in the 1^st^, 2^nd^, 3^rd^, and 4^th^ convolution layer, respectively. The number of filters in each layer is 36. The batch size is 20% of the training data. In addition, the CNN model has been trained in 1000 epochs. In CNN, the feature extraction phase is automatically performed by convolution layers; therefore, the CNN model does not requires the feature computation done by the three employed image texture descriptors.

**Figure 8:**
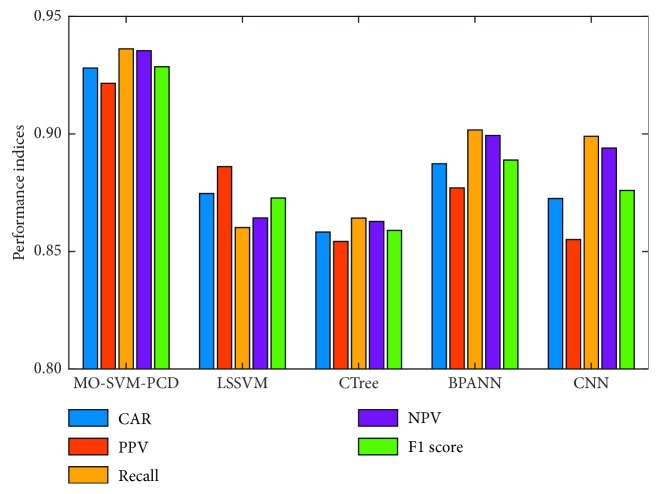
Result comparison.

The prediction results of all the models obtained from the repeated data sampling with 20 runs are summarized in Table 3 which reports the mean and the standard deviation (Std) of the model performance. Observably, the MO-SVM-PCD has attained the most desired predictive accuracy in terms of CAR, followed by BPANN, LSSVM, CNN, and CTree. The proposed pipe corrosion approach also achieves the highest values of PPV, recall, NPV, and F1 score. The comparison of model performance is graphically displayed in Figure 8.

In addition, the Wilcoxon signed-rank test [60] is utilized in this section to better confirm the statistical significance of the differences in the model performances. This is a nonparametric statistical test commonly employed for model comparison [61]. With the significance level of the test = 0.05, if the *p* value computed from the Wilcoxon signed-rank test is lower than this significance level, it is able to reject the null hypothesis of insignificant difference in prediction outcomes of the two predictors. Hence, it is confident to conclude that the predictive results of the two pipe corrosion detection models are statistically different. Using the CAR values, the outcome of the Wilcoxon signed-rank tests is reported in Table 4. This test points out that the MO-SVM-PCD is statistically better than the LSSVM, CTree, BPANN, and CNN with *p* values < 0.05. Based on this statistical test, it is able to state that the proposed method is the most suited method for the task of interest.

## 4. Conclusion

Corrosion is a commonly observed type of pipe defects. Timely detection of corrosion is very crucial to ensure the integrity of the water supply system and avoid water contamination. In addition, information regarding corroded pipe sections obtained during periodic building surveys can significantly help to establish cost-effective maintenance strategies for building owners. This study puts forward an automatic method based on image processing and machine learning for pipe corrosion recognition. Image processing techniques have been employed to extract useful features from images of pipe surface to characterize the corrosion status. In total, 78 features are extracted using three texture descriptors of the statistical properties of image color, GLCM, and GLRL.

The SVM machine learning method integrated with the DFP metaheuristic is utilized to construct a decision boundary used for classifying pipe surface images into two categories of noncorrosion and corrosion. A dataset consisting of 2000 image samples has been used to train and validate the proposed hybrid model of the MO-SVM-PCD. Experimental results supported by the Wilcoxon signed-rank test point out that the newly developed method is superior to other benchmark approaches with an average CAR = 92.81%. Therefore, the newly developed model can be a useful tool for building maintenance agents to quickly evaluate the status of pipe systems. Further extensions of the current study may include the utilization of other advanced machine learning for data classification, employment of other metaheuristic for model optimization, employment of higher-order statistical features as input to machine learning based classifiers, enhancement of the detection accuracy for image samples located in the boundary of the corroded area, improvement of the computational efficiency of the current method by employing advanced image segmentation techniques, and collection of more image samples to enhance the generalization of the current MO-SVM-PCD model.

## Figures and Tables

**Table 1:** Texture descriptors using GLRL.

Descriptor	Notation	Equation
Short run emphasis	SRE	SRE=1/*N*_r_∑_*i*=1_^*M*^∑_*j*=1_^*N*^ *p*(*i*, *j*)/*j* ^2^
Long run emphasis	LRE	LRE=1/*N*_r_∑_*i*=1_^*M*^∑_*j*=1_^*N*^ *p*(*i*, *j*) × *j* ^2^
Gray-level nonuniformity	GLN	GLN=1/*N*_r_∑_*i*=1_^*M*^(∑_*j*=1_^*N*^ *p*(*i*, *j*)^2^)
Run length nonuniformity	RLN	RLN=1/*N*_r_∑_*j*=1_^*N*^(∑_*i*=1_^*M*^ *p*(*i*, *j*)^2^)
Run percentage	RP	RP=*N*_r_/*N*_p_
Low gray-level run emphasis	LGRE	LGRE=1/*N*_r_∑_*j*=1_^*N*^∑_*i*=1_^*M*^ *p*(*i*, *j*)/*i* ^2^
High gray-level run emphasis	HGRE	HGRE=1/*N*_r_∑_*j*=1_^*N*^∑_*i*=1_^*M*^ *p*(*i*, *j*) × *i* ^2^
Short run low gray-level emphasis	SRLGE	SRLGE=1/*N*_r_∑_*j*=1_^*N*^∑_*i*=1_^*M*^ *p*(*i*, *j*)/(*i* ^2^ × *j* ^2^)
Short run high gray-level emphasis	SRHGE	SRHGE=1/*N*_r_∑_*j*=1_^*N*^∑_*i*=1_^*M*^(*p*(*i*, *j*) × *i* ^2^)/*j* ^2^
Long run low gray-level emphasis	LRLGE	LRLGE=1/*N*_r_∑_*j*=1_^*N*^∑_*i*=1_^*M*^(*p*(*i*, *j*) × *j* ^2^)/*i* ^2^
Long run high gray-level emphasis	LRHGE	LRHGE=1/*N*_r_∑_*j*=1_^*N*^∑_*i*=1_^*M*^ *p*(*i*, *j*) × *i* ^2^ × *j* ^2^

*Note*. *M* and *N* are the number of gray levels and the maximum run length, respectively. Let *N*_r_ and *N*_p_ be the total number of runs and the number of pixels in the image, respectively.

**Table 2:** Average prediction performance of the MO-SVM-PCD.

Indices	Training phase	Testing phase
CAR (%)	98.382	92.808
PPV	0.982	0.922
Recall	0.986	0.936
NPV	0.986	0.935
F1 score	0.984	0.929

**Table 3:** Corrosion detection result of the machine learning models.

Phase	Indices	MO-SVM-PCD		LSSVM		CTree		BPANN		CNN	
		Mean	Std	Mean	Std	Mean	Std	Mean	Std	Mean	Std
Train	CAR (%)	98.382	0.236	96.432	0.435	97.018	0.532	94.593	1.674	90.890	1.649
	PPV	0.982	0.004	0.937	0.008	0.970	0.006	0.937	0.020	0.891	0.024
	Recall	0.986	0.004	0.996	0.002	0.971	0.008	0.956	0.016	0.933	0.020
	NPV	0.986	0.004	0.995	0.002	0.971	0.007	0.956	0.016	0.930	0.019
	F1	0.984	0.002	0.965	0.004	0.970	0.005	0.947	0.016	0.911	0.016

Test	CAR (%)	92.808	1.094	87.467	1.121	85.825	1.467	88.733	1.721	87.258	1.571
	PPV	0.922	0.015	0.886	0.016	0.854	0.016	0.877	0.022	0.855	0.028
	Recall	0.936	0.017	0.860	0.016	0.864	0.021	0.902	0.026	0.899	0.022
	NPV	0.935	0.016	0.864	0.014	0.863	0.019	0.899	0.024	0.894	0.018
	F1	0.929	0.011	0.873	0.011	0.859	0.015	0.889	0.017	0.876	0.014

**Table 4:** *p* values obtained from the Wilcoxon signed-rank test.

Models	MO-SVM-PCD	LSSVM	CTree	BPANN	CNN
MO-SVM-PCD	—	0.00009	0.00009	0.00009	0.00009
LSSVM	0.00009	—	0.00427	0.01407	0.48933
CTree	0.00009	0.00427	—	0.00022	0.01185
BPANN	0.00009	0.01407	0.00022	—	0.01954
CNN	0.00009	0.48933	0.01185	0.01954	—

## Data Availability

The data used to support the findings of this study are available from the corresponding author upon request.

## Supplementary Materials

Supplementary Materials The supplementary material of this study contains the dataset used to construct the machine learning-based model. The first 78 columns of the dataset are the extracted texture features. The last column is the class label (0 for noncorrosion and 1 for corrosion).
